# Selenium in Proteins: Conformational Changes Induced by Se Substitution on Methionine, as Studied in Isolated Model Peptides by Optical Spectroscopy and Quantum Chemistry

**DOI:** 10.3390/molecules27103163

**Published:** 2022-05-15

**Authors:** Gildas Goldsztejn, Venkateswara Rao Mundlapati, Valérie Brenner, Eric Gloaguen, Michel Mons

**Affiliations:** Université Paris-Saclay, CEA, CNRS, LIDYL, 91191 Gif-sur-Yvette, France; gildas.goldsztejn@universite-paris-saclay.fr (G.G.); mvraochem2008@gmail.com (V.R.M.); valerie.brenner@cea.fr (V.B.); eric.gloaguen@cea.fr (E.G.)

**Keywords:** seleno-methionine, H-bonding, side-chain–backbone interactions, gas phase, conformational analysis, conformation-selective spectroscopy, laser, quantum chemistry

## Abstract

The side-chain of methionine residues is long enough to establish NH⋯S H-bonds with neighboring carbonyl groups of the backbone, giving rise to so-called intra-residue 6^δ^ and inter-residue 7^δ^ H-bonds. The aim of the present article is to document how the substitution of sulfur with a selenium atom affects the H-bonding of the Met system. This was investigated both experimentally and theoretically by conformation-resolved optical spectroscopy, following an isolated molecule approach. The present work emphasizes the similarities of the Met and Sem residues in terms of conformational structures, energetics, NH⋯Se/S H-bond strength and NH stretch spectral shifts, but also reveals subtle behavior differences between them. It provides evidence for the sensitivity of the H-bonding network with the folding type of the Sem/Met side-chains, where a simple flip of the terminal part of the side-chain can induce an extra 50 cm^−1^ spectral shift of the NH stretch engaged in a 7^δ^ NH⋯S/Se bond.

## 1. Introduction

Selenium is essential to the living world [1,2]. Despite chemical and physical properties close to those of sulfur, namely similar outer valence-shell configuration and electron affinities, nature has taken advantage of its specific properties, in particular its redox properties, to provide proteins with resistance mechanisms to permanent oxidation [2]. In proteins, Se is thus present in two residues, Se-cystein, one of the proteinogenic amino acids, and the non-genetically coded seleno-methionine (Se-Met or, in short, Sem) [1]. The latter is randomly incorporated in proteins due to several factors: first, it mimics the chemical properties of the sulfur-based amino acid methionine (Met), preventing the genetic code machinery to discriminate between Met and Se-Met [3]; second, methionine is an essential amino acid, taken from the diet, and finally, seleno-methionine is abundant in some plant-originated food, such as Brazil nuts, grassland legumes and soybeans [1,4], although with marked fluctuations depending upon the crop region [5]. This leads to an isosteric replacement of sulfur by selenium, which is thought to have little effect on the structure [1], for instance, the existence of such heavy atom mutants has been widely used by physicists to improve structural information, e.g., by facilitating the phasing problem in X-ray crystallography [1,6].

Despite the expected similarities between chalcogens, Se differs from S with a few features, potentially critical for structural issues, namely its van der Waals radius, its polarisability and nucleophilic properties [1,2]. Se is also often thought of as a weaker nucleophile and thus a weaker H-bonding acceptor [1,2]. These views, however, were recently questioned [7,8,9], especially in view of studies on model thio- and seleno-compounds, led under isolated conditions, which provided evidence for strong interactions of S and Se heteroatoms with NH groups; information considered as transferable to proteins [9]. Optical IR and UV spectroscopic studies of model systems of biological relevance, indeed, have recently targeted the S heteroatom [9,10,11,12,13] and more recently selenium [9,14,15]. In most cases, various models of biomolecules are complexed with a dimethylsulfide or dimethylselenide, which enables modeling the acceptor H-bonding properties of the chalcogen divalent heteroatom.

**Scheme 1:**
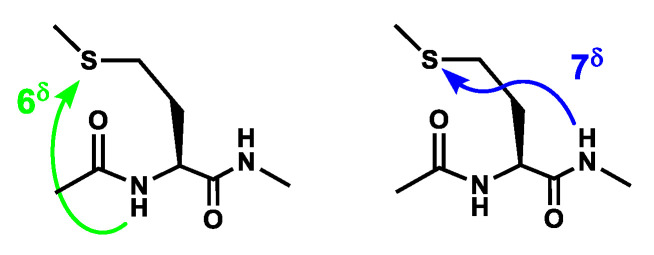
Local side-chain—backbone interactions of a Met side-chain.

**Scheme 2:**
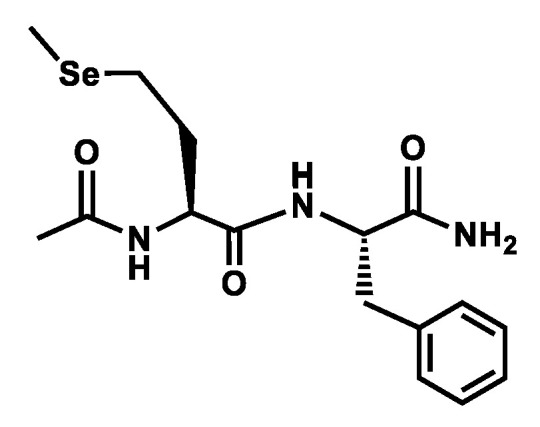
Model compound studied.

However, experimental data on directly relevant models of protein chains such as amino acids or peptides remain rare. Although S-based side-chain–backbone local H-bond interactions were documented with Cys [16,17,18] and Met [19] (Scheme 1), only experimental results on Se-Cys [18] were recently reported together with a Protein Data Bank survey suggesting the occurrence of strong H-bonding with Se [9]. The aim of the present work is to document the local interactions of the Se heteroatom of a Se-Met residue in a two-residue model of a protein chain. Taking a gas phase approach, the conformational diversity and H-bonding content of a model of peptide chain containing a Se-Met residue, namely Ac-Sem-Phe-NH_2_ (Scheme 2), were probed using IR and UV state-of-the-art spectroscopic diagnostics and assigned by comparison with quantum chemistry calculations [20,21]. The presence of the Phe residue in the sequence is justified by the need for a near-UV chromophore in the system in order to perform IR-UV spectroscopy. The chemical protection of the Sem-Phe sequence was chosen in order to avoid any side effects due to ends that would not be relevant to a protein chain, and thus to properly mimic a segment of such a chain, i.e., containing complete -CONH- peptide bonds.

The study focuses on the understanding of the backbone folding (the presence of C5, C7 or C10 H-bonds, stabilizing, respectively, extended, γ-turn or β-turn structures) in these model backbones together with its interplay with the local H-bonding interactions that Se can establish with the backbone NH groups, namely the so-called 6^δ^ intra-residue or 7^δ^ inter-residue main chain–side-chain H-bonds [22]. Despite their local character, these bonds (Scheme 1) are expected to sample, at least partially, the diversity of NH⋯Se interactions encountered in proteins. A comparison of these results with those on the related sulfur compound (Ac-Met-Phe-NH_2_) [19] is also carried out to document the specificities of Se relative to S as well as the effect of the long side-chain of methionine.

## 2. Methods

The gas-phase spectroscopy of the peptide samples was recorded using an experimental set-up and procedures described elsewhere [21,23]. Sample vaporization was carried out using homemade laser-desorption equipment, based on a pulsed valve, generating gas pulses, which expanded in an efficiently pumped vacuum chamber (pressure of 2 × 10^−5^ torr during operation). A solid pellet, made of pressed powder of the peptide of interest (Genscript, USA) mixed with graphite, was placed at the exit nozzle (1 mm diameter) of the pulsed valve, operating with a 30:70 mixture of Ne:He at a backing pressure of 20 bars. The output of a frequency-doubled YAG laser (0.1–2 mJ/pulse) was sent through an optical fiber on the surface of the pellet, causing the sample vaporization. Laser-desorbed molecules were then entrained and cooled down by the pulsed supersonic expansion, and after passing through a skimmer, entered the extraction region of a time-of-flight mass spectrometer, where optical interaction with pulsed ns lasers took place. The resulting ions were then detected on a microchannel plate detector, according to their m/z mass channel. The YAG-pumped dye laser (NarrowScan, Radiant Dyes Laser GmbH, Germany) was scanned to record mass-selected UV spectra, through resonant two-photon ionization (R2PI). Conformer-selective IR spectroscopy in the NH stretch region was obtained through double resonance IR/UV spectroscopy: the IR laser (OPO/OPA, LaserVision, USA) was scanned whereas the UV laser was tuned to a transition of the desired conformer, leading to a depletion of the ion signal when this specific conformer absorbed the IR light. The IR spectra were obtained by comparing UV signals with IR laser on and off, according to a procedure described previously [23].

The IR spectra obtained are then compared to simulated spectra obtained for the most stable conformations of the peptide. Regarding theoretical calculations, conformational exploration was carried out using the OPLS_2005 force field model within the Macromodel suite [24], followed by a quantum chemistry level optimization carried out using the TurboMole package [25] at the DFT-D level of theory (RI-B97-D3(BJ-abc)/def2-TZVPPD), which was proved to be relevant for the conformation description of gas-phase peptides [21,26]. Among the numerous conformations found by the molecular mechanic calculation, the candidates that were optimized at the DFT level of theory—approximately a hundred—were chosen firstly on the ground of relative energies given by the force field, and secondly as representative of different families of structures, using a home-made script to regroup the conformers in structural clusters. The latter point was important in order to make sure that the DFT calculations were representative of the whole conformational landscape.

Theoretical energetics were obtained at both 0 K and 300 K, the latter being usually relevant for describing the conformational distribution of desorbed peptides [21,27]. The theoretical gas-phase spectra were then obtained from the theoretical harmonic frequencies, once scaled using mode-dependent scaling factors, calibrated from linear fits of the correlation between experimental and harmonic NH stretch frequencies (f^scaled^ = a + b × f^harmonic^) on a large library of gas-phase peptides (cf. Supp. Info. of Ref. [28]). The scaling factors (a, b) applied to the harmonic frequencies were recalculated for the level of theory presently used: for a peptide NH stretch mode: a = 372.8 cm^−1^ and b = 0.86953; for a carboxamide NH_2_ symmetric stetch mode, a = 1209.8 cm^−1^ and b = 0.63115 and for its antisymmetric counterpart, a = 1324.1 cm^−1^ and b = 0.60872. This procedure usually provides a typical accuracy of 20 cm^−1^ in the NH stretch range [26]. The assignment is eventually derived from the best fit between experimental spectra and those of the most stable peptide structures calculated at 300 K [26].

Finally, a Natural Bonding Orbital (NBO) analysis of the NH⋯S/Se H-bonding interactions was carried out to quantify the stabilizing role of electron delocalization through H-bonding effects on remarkable conformations of the capped Sem-Phe and Met-Phe dipeptides studied as well as on the (thio/seleno-dimethylether⋯*trans*-methylacetamide; in short S/Se(Me)_2_⋯ MAA) intermolecular model reference system, in a similar way as in a previous study on the amide–amide interactions in peptides [29]. Calculations [30,31,32] were performed on the RI-B97-D3(BJ)-abc/TZVPPD structures using the NBO module [33] of the Gaussian 16 software [34], following a procedure described in detail previously [22,29].

In order to characterize the NH⋯Se/S H-bonding in relevant conformations, the stabilization energies resulting from the electron delocalization between NBOs of the H-bond proton acceptor (Se) and donor (NH), i.e., the donating NBO (Lewis-like occupied lone pairs of the Sem/Met side-chain Y atom, Y = S or Se, labeled n_Y_ and n’_Y_) and the accepting unoccupied σ_NH_* NBO, respectively, were summed up to provide the total stabilization energy, noted ΣE_HB_, due to the H-bond considered.

## 3. Conformational Landscapes

**Figure 1:**
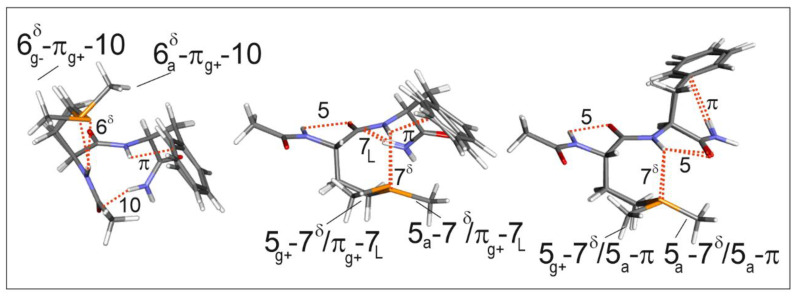
Typical low energy conformations of the folded (left), semi-extended (center) and extended (right) conformational families of Ac-Sem-Phe-NH_2_, as obtained at the RI-B97D3(BJ-abc)/def2-TZVPPD level of theory (see text for terminology details). The folded and semi-extended/extended forms are shown with a *gauche +* and *anti* Phe side-chain, respectively (labeled by the second subscript), which corresponds to their most stable conformation. The family of semi-extended forms, illustrated here by the *gauche+* orientation, is much richer since alternative backbone and side-chain orientations can also be energetically competitive; also see text for the terminology used. Note here that for each conformation, the Sem side-chain can exhibit two terminal orientations (labeled by the first subscript).

The exploration of the conformational landscape of Ac-Sem-Phe-NH_2_, coupled with quantum chemistry structure optimizations, led to two types of structures: either, overall folded forms based on the presence of a β-turn; stabilized by a C10 H-bond, or, more or less extended forms, based on C5 or C7 backbone local interactions, where the two ends of the molecule do not interact with each other (Figure 1).

- The folded backbone forms are additionally stabilized by a side-chain/main chain intra-residue H-bond, forming a 6-membered ring, where the side-chain heteroatom Se plays the role of proton acceptor. According to the terminology used previously [21], based on the H-bonding status of the successive NH bonds along the backbone, these conformations belong to a 6^δ^-π-10 folded backbone family: the number stands for the length of the ring formed by the H-bond concerned, the δ superscript for a backbone–side-chain H-bond bridging an NH bond with a side-chain acceptor in a δ position relative to the main chain, and π for an NH–π interaction with Phe ring. The structural analysis shows that the intra- residue 6^δ^ H-bond can take place with a specific side-chain folding, described by a *gauche+* (*g*+) and *gauche-* (*g*−) orientation of the χ_1_ and χ_2_ dihedrals (i.e., the N-C^α^-C^β^-C^γ^ and C^α^-C^β^-C^γ^-Se dihedrals), respectively. Two possible orientations of the terminal methyl group of the Sem side-chain (described by the C^β^-C^γ^-Se-C^ε^ χ_3_ dihedral) can take place: *gauche−* and *anti* (*a*), respectively, which give rise to significantly different H-bond lengths and strengths (see below). For the sake of simplicity, the Sem side-chain conformation is thus identified by the orientation of the terminal group (*g*− or *a*). Similarly, the Phe side-chain can take three possible orientations (*g*+, *g*− or *a*) depending on the χ_1_ dihedral, but in the present case, *g*+ conformations are favored by the formation of an intra-residue NH–π H-bond. For the sake of identification, conformations have thus been labeled with the Sem and Phe side-chains’ orientations, given as indices on the symbol (5, 7, 6^δ^, 7^δ^, π, or f (for free)) that indicates the H-bonding status of the NH group of the corresponding residue.
  The extended and semi-extended forms correspond to the succession of local conformational preferences of the basic peptide backbone, found with Ala or residues, i.e., the so-called C5 or C7 structures corresponding to β-sheet or γ-turns, respectively, the latter folding existing under two chiral forms, the inverse and direct γ-turns, labeled L or D, respectively. The key point here is the fact that the presence of the Sem side-chain does locally stabilize an extended C5 backbone conformation, thanks to the formation of a 7^δ^ inter-residue H-bond. Again the Sem side-chain end adopts conformations where such a bond can form (χ_1_ and χ_2_ with *a* and *g*+ orientations, respectively), but the orientation of the terminal methyl group of the Sem side-chain (described by the χ_3_ dihedral) can occur either in an *a* or a *g*+ orientation. As a consequence, the semi-extended and extended forms (Figure 1) exhibit a 5-7^δ^-7 and a 5-7^δ^/5-π backbone, respectively, the 7^δ^/5 notation standing for a second NH group simultaneously involved in 7^δ^ and C5 interactions. Finally, depending on the Phe side-chain orientation, additional π H-bonds can form. In semi-extended forms with a Phe *g*+ orientation, the second NH group is simultaneously involved in a 7^δ^ H-bond and a π interaction with the Phe side-chain (notation 7^δ^/π_g+_ in Figure 1), without strongly affecting the overall backbone structure, the 7^δ^ H-bonds and their relative strengths.

**Figure 2:**
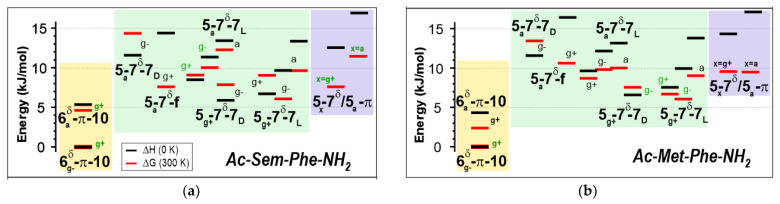
Conformational landscape of Sem-Phe (left panel; (**a**)) and Met-Phe (right panel; (**b**)) peptide chains, obtained at the RI-B97-D3(BJ-abc)/def2-TZVPPD level of theory. The 0 K ΔH relative energies (resp. 300 K Gibbs energies, ΔG) are given as black (resp. red) lines. The conformation labels refer to the sequential status of the N–H bonds along the backbone (see text). The *g*+/*g*−/*a* subscripts of the folded and semi-extended conformations refer to the χ_3_ dihedral of the Sem/Met side-chain; the labels associated with the bar of each conformation refer to Phe side-chain χ_1_ orientation (*g*+/*g*−/*a*). In the extended conformation family, the x label refers to the Sem/Met side-chain χ_3_ dihedral. Green labels indicate conformations, which are consistent with experimental observations.

Regarding the relative stability of these three families (Figure 2), the β-turn family is by far the most stable one compared to the extended and semi-extended forms. The latter is, however, much richer because, in contrast to the other families, different Phe orientations can lead to comparable stabilities, due to various opportunities for interactions involving the Phe side-chain with other parts of the molecule. Additionally, related structures differing by the absence of one of the interactions (and hence entropically favored) or the presence of an alternative form (7_D_ H-bond instead of 7_L_) tend to enhance the family population at high (room) temperature, which was found to be a relevant temperature to describe the conformational population in the supersonic expansion (due to the rapid conformational freezing taking place in it) [27,28].

## 4. Experimental Results and Assignments

### 4.1. UV Spectroscopy

**Figure 3:**
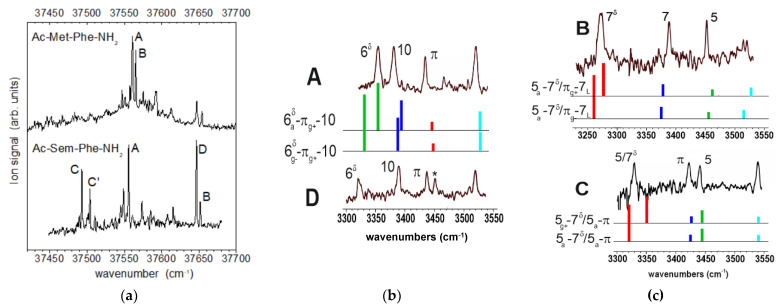
(**a**) left panel: Near UV spectrum of the Ac-Sem-Phe-NH_2_ molecule in the origin region of first ππ*transition of Phe, as obtained by mass-selected R2PI. *Top*: For comparison, the same spectrum for the related compound with Met is also shown (adapted with permission from Ref. [19]; Copyright 2012 American Chemical Society). (**b**,**c**) central and right panels: IR/UV double resonance spectra of conformers A–D (black traces), compared to the theoretical spectra (colored sticks) of selected best matching, low energy conformations (see Figure 2): A and D are assigned to folded 6*^δ^*-*π*-10 conformations; B and C to extended 5-7*^δ^*/5-7 forms. The IR experimental spectra (A–D) were recorded by probing the UV band bearing the same label in Figure 1. The asterisk in spectrum D suggests a secondary contribution to the IR spectrum (see text for discussion). The color code of the stick spectra (green, red, blue and cyan bars) corresponds to NH stretches of the Sem, Phe residues and of the symmetric and antisymmetric C-terminal amide, respectively.

The near UV spectrum of the Ac-Sem-Phe-NH_2_ compound, obtained using the resonant two-photon ionization technique, was composed of several narrow bands (Figure 3a) ranging over more than 150 cm^−1^. Noticeably, it presented an intense band, located at 37,556 cm^−1^ (A band), and doublets around 37,650 cm^−1^ (D and B bands) and 37,500 cm^−1^ (C and C′). Owing to the similarity expected between Met and Sem dipeptides, the spectrum was compared with that of the Met-related dipeptide, namely Ac-Met-Phe-NH_2_, for which a doublet of UV features, labeled A and B (Figure 3a), was previously [19] assigned to a folded 6^δ^-π-10 β-turn form (A) and a semi-extended 5-7^δ^-7 structure (B), corresponding to different H-bonding of the Met side-chain to the backbone. Interestingly, the band A of the Sem molecule is located in the same range as the A-B UV doublet of Ac-Met-Phe-NH_2_. However, owing to the proximity of the doublet lines, belonging to different conformations, it appears difficult to infer an assignment out of sole UV data. More interesting, the D-B doublet seems to have a counterpart in the Met molecule, but D appears to be a more intense band in the Sem molecule. Finally, the C-C′ doublet does not show any counterpart in Met. This relatively red-shifted position is strikingly close to that observed for the extended forms of Ac-Phe-NH_2_ [35], Ac-Ala-Phe-NH_2_, Ac-Gly-Phe-NH_2_ [36], Ac-Cys-Phe-NH_2_ [17] and Ac-Sec-Phe-NH_2_ [22] molecules, suggesting a conformational population with Sem differing from that observed with Met. In support of this, we observed that the C-C′ doublet is found to appear in hot conditions of the supersonic expansion, more precisely, at short delays between the pulsed valve aperture and the desorption laser pulse.

### 4.2. IR Spectroscopy

#### Conformational Population of Ac-Sem-Phe-NH_2_

In order to access the conformational and structural content underlying the UV spectra, double resonance IR/UV experiments were carried out to cover the NH stretch region, by probing each of the intense (labeled) bands of the UV spectrum in Figure 3a. The IR spectra obtained (Figure 3b,c) turned out to exhibit different natures. Whereas four NH stretch bands are expected due to the four NH oscillators of the molecule (as illustrated by the IR spectra of the Met compound [19]), which are observed in UV bands A, B and C, the intense D band showed a more complex IR pattern, with one extra band at 3452 cm^−1^ (marked with an asterisk in Figure 3b), which was eventually interpreted as a hybrid IR spectrum composed of the main component (D conformer) superimposed with a minor additional population (whose nature will be discussed below). In addition, the IR spectrum obtained from C′, which is identical to that from C, C′, was assigned to a vibronic band at 12 cm^−1^ from the C origin. As a result, only four different IR spectra were found (Figure 3) demonstrating the existence of four different conformers, populated in the expansion.

Taking advantage of the absence of coupling between the NH stretch modes of the molecules, interesting qualitative information was readily derived from the examination of IR spectra. In each spectrum, the position of the vibrational bands reveals the type of interaction undergone by the corresponding NH bonds [21]: below 3400 cm^−1^ an H-bond (7^δ^, 6^δ^, or C10) in the 3420–3460 cm^−1^ weak interactions such as C5 or π bonds, and around 3530 cm^−1^, the asymmetric stretch band of NH_2_. A careful examination showed a marked resemblance of A and D IR spectra (provided that the band of the D spectrum that is marked with an asterisk is discarded). The main difference stems from the strength of the strongest H-bond, suggesting a shared backbone structure. In contrast, B and C showed different patterns, as testified by the spectral position of the antisymmetric NH_2_ stretch. In B, the asymmetric NH_2_ stretch band appears as a weak, noisy band at 3520 cm^−1^, which revealed an H-bond engaged NH, whereas the blue position at 3540 cm^−1^ in C suggested the involvement in a significantly weaker interaction, typically a π-type. Moreover, B exhibited the strongest H-bond of all the observed forms.

For a more detailed analysis, the experimental spectra were compared with the theoretical IR spectra of the most stable forms obtained in the conformational analysis of Ac-Sem-Phe-NH_2_ (Figure 2). Following a procedure successfully used previously [23], the theoretical spectra obtained for the most stable forms of each family at 300 K (see Supplementary Materials: Table S1) were compared to the experimental spectra and assessed according to the RMS and maximum unsigned error to the experimental bands.

The examination shows that in most cases, quite a good agreement was met for one of these low energy structures and that, in most of the cases, it was possible to distinguish between *a* and *g*+ Sem side-chain orientations.

The good agreement of A and D spectra with those of the two most stable forms of the 6^δ^-π-10 conformational family (RMS and unsigned maximum errors lower than 8 and 13 cm^−1^ resp.; see Table S1) provided a straightforward assignment of A and D to β-turn structures (6^δ^_a_-π_g+_-10 and 6^δ^_g−_-π_g+_-10), with *a* and *g*− χ_3_ Sem side-chains, respectively (Figure 3b). The *g*+ side-chain in D provided a slightly better 6^δ^ H-bond approach and distance than the *a* form (248 pm vs. 251 pm) as testified by the spectral red-shift of ~20 cm^−1^ for the corresponding IR band relative to A. In both cases, the orientation of the Phe side-chain (*g*+) enables the formation of a π H-bond. Satisfactorily, conformer A exhibits a UV frequency (37,557 cm^−1^) that matches that of such a β-turn in the absence of any other group interacting with Phe (Cf. the transition of the β-turn B of Ac-Gly-Phe-NH_2_ at 37,562 cm^−1^ [36]). The blue shift of the band of conformer D should then be ascribed to the interaction between the Se lone pairs and the Phe ring, which are facing each other in this structure.

At this stage, the origin of the asterisk-marked additional band in the IR spectrum D at 3452 cm^−1^ should be discussed. It is located in the region of weakly H-bonded NHs, between π-bonded and free NHs [26]. Several hypotheses can be put forward: (i) the occurrence of Fermi resonance between the NH mode involved in the π H-bond and a CO stretch mode in the 1730 cm^−1^ region, the latter being, however, not confirmed by calculations, or (ii) it may arise from another oscillator, e.g., an H-bonded OH stretch of a water molecule; its presence in the IR spectrum would be due to the presence of a hydrate of monomer D in the supersonic expansion: if a water molecule solvates an acceptor site located far from the UV chromophore (e.g., the Se atom), the UV features of the complex can coincide with those of the corresponding monomer, its IR bands are expected to differ essentially by the water bands, the symmetrical OH stretch is possibly located in the region considered [13,28]. Finally, since post-photoionization fragmentation is often encountered in these systems [28], the hydrate’s signatures are anticipated in the monomer mass channel.

Conformer B exhibited a different IR pattern (Figure 3c), which was well accounted for by two conformations among the most stable ones within the 5-7^δ^-7 backbone family at 300 K (see Table S1), namely the 5_a_-7^δ^_g+_-7_L_ and 5_a_-7^δ^_g*−*_-7_L_ forms. The red shift of the 7^δ^ band indicates unambiguously an *a* χ_3_ dihedral for Sem, leading to an NH⋯Se approach with a short distance of 259 and 249 pm for *g*+ and *g*− Phe side-chains, respectively. It can be noted that despite the 7^δ^NH⋯Se distances being comparable to or longer than the 6^δ^ H-bonds, the resulting spectral shifts indicate a stronger H-bonding, suggesting that the 7^δ^ H-bond benefits from a much more efficient geometrical approach [29] than its 6^δ^ counterpart. This point will be further discussed in Section 5.3.

A further assignment in terms of Phe side-chain orientation (*g*+ vs. *g*−) is difficult to propose. It is nevertheless interesting to notice that the semi-extended form with a 7_D_ H-bond (5_a_-7^δ^_g−_-7_D_) that also provides a fair spectral match can be discarded due to its unfavorable energetics.

In contrast, for the C form, a good match is found for the lowest 5_a_-7^δ^/5_a_-π extended backbone conformation, despite a relatively surprising unfavorable energetics (ΔG of 7.6 kJ/mol; see Table S1). Again the red shift of the 7^δ^ band pleads in favor of an *a* orientation of the Sem side-chain χ_3_ over its *g*+ challenger, this latter being additionally still higher in energy at a low temperature (Figure 2). The assignment to an extended form, at least on the Phe residue, is supported by the remarkable UV spectral features of C: the *a* Phe side-chain makes the structure around Phe very similar to that of the main conformer β_L_(*anti*) of the Ac-Phe-NH_2_ (NAPA) molecule, accounting for both the range of its UV transition (cf. 34,500 cm^−1^ in NAPA) and its Franck–Condon activity, namely the presence of the C′ band assigned to a vibronic band, associated to an active low-frequency mode in the S_1_ state (18 cm^−1^ in NAPA).

## 5. Discussion

### 5.1. Revisiting the Spectroscopy of Ac-Met-Phe-NH_2_

The present results on the seleno-methionine capped dipeptide were also an opportunity to revisit previous investigations on its sulfur equivalent. For this purpose, a landscape investigation was also carried out at the same level of theory as for the Sem compound. The results (Figure 2) illustrate the striking similarities between these conformational spaces, which share the same three backbone family patterns. They differ essentially by minor energetics changes, the extended forms appearing slightly more unfavoured in Met than in Sem dipeptides, as well as the semi-extended forms 5_a_-7^δ^-7_L_, with an *ag+a* Met side-chain.

IR spectra were also calculated at the same level of theory; satisfactorily the results were qualitatively comparable to those obtained previously [19] at a more modest level of theory for a more limited set of conformations, but seemed to achieve a better match to the experiment (see Table S2).

**Figure 4:**
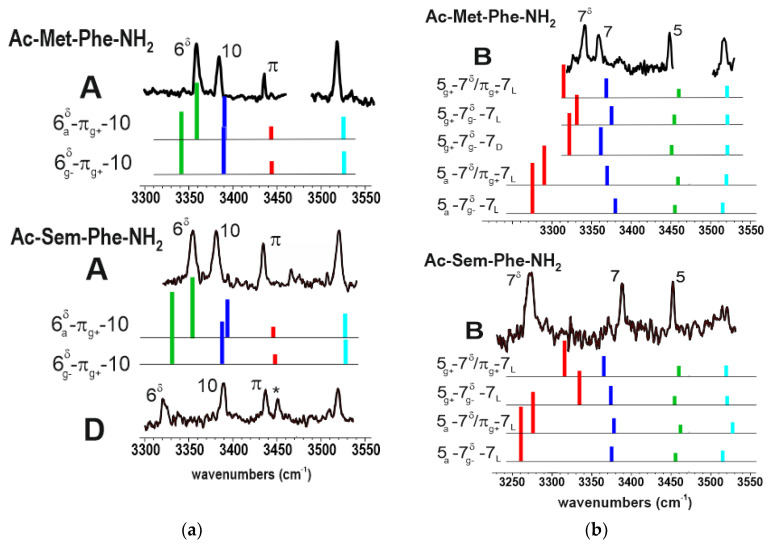
(**a**) left panel: IR/UV double resonance spectra of folded conformers observed in the Sem-Phe and Met-Phe sequences, compared to the theoretical spectra (colored sticks) of selected best matching, low energy conformations. The comparison illustrates the similarities of the 6^δ^ H-bonds in these sequences; (**b**) right panel: same pictures for the semi-extended conformers B, emphasizing the apparent differences in the 7^δ^ H-bonds between the two sequences. See text for assignment.

Figure 4a compares the IR spectra of conformations assigned to a β-turn (6^δ^-π-10 family), namely conformation A of the MetPhe sequence, and A and D for the Sem-Phe sequence, with relevant theoretical spectra. The experimental spectra of conformations A present marked similarities, suggesting the same assignment; a conclusion partly supported by the slightly better agreement met for the 6^δ^_a_-π_g+_-10 form, although its challenger (6^δ^_g+_-π_g+_-10) would not have been rejected on sole theoretical grounds (see Table S2). The band A of the Met-Phe sequence, previously assigned to the 6^δ^_g+_-π_g+_-10 structure by Biswal et al. [19] (conf a in this reference), is eventually assigned to the 6^δ^_a_-π_g+_-10 form. Interestingly, UV data (Figure 3a) provide additional information: they support the assignment since in both sequences the band A is found in the same spectral region (37,560 cm^−1^) according to the weak substitution effect expected. Additionally, it also suggests a prediction: the band at 37,650 cm^−1^ with Met (not investigated in [19]), which coincides with the D band of Sem, can be tentatively assigned to the 6^δ^_g+_-π_g+_-10 form of this molecule.

In passing, here, one will notice that the *g+g−g−* Met-Sem side-chain gives rise to a slightly stronger 6^δ^ H-bond. This point will be further discussed later on.

Regarding the semi-extended form (conformer B) of the Met-Phe sequence, it was previously assigned to the 5_g+_-7^δ^_g*−*_-7_D_ conformation by Biswal et al. [19]. This form, as one of the most stable semi-extended forms (Figure 2), is indeed one possibility, but alternative assignments, with comparable energetic and IR fitting scores, are also possible, namely the 5_g+_-7^δ^/π_g+_-7_L_ and 5_g+_-7^δ^_g−_-7_L_ forms (See Figure 4b and Table S2). Concerning the absence of such a 5_g+_-7^δ^-7_L_ form in the Sem-Phe sequence, energetic data at 300 K (Figure 2) suggest that such backbone with Sem is less stabilized relative to its 5_a_-7^δ^-7_L_ counterpart with Met. It could be that these forms correspond to the weak (not probed) UV bands in the vicinity of band A in the Sem-Phe spectrum of Figure 3a.

As far as the extended form C is concerned, its absence in the Met-Phe sequence [19] should probably be ascribed to a less complete exploration of the experimental conditions in this early study. As a matter of fact, depending on the expansion conditions chosen when recording the UV spectra, the C-C′ bands can be easily overlooked.

### 5.2. Comparing the H-Bonding Patterns of Sem and Met

The striking similarity of the IR spectra of bands A of the Sem-Phe and Met-Phe sequences (Figure 4a) suggests close H-bonding features in these conformations, in particular very similar 6^δ^ H-bond strengths for S and Se heteroatoms, with a slight advantage to Se, which exhibits, for the same conformation, a red shift increase of ~15 cm^−1^. As far as the B bands are concerned (Figure 4a), however, the situation seems to be much more contrasted. Whereas the C7 and C5 backbone H-bond features remain unchanged (C5) or in the same region (within 30 cm^−1^ for C7), it is no longer the case for the 7^δ^ bonds, whose spectral shifts differ by typically 65 cm^−1^. While one could be tempted to assign the observation to a specific property of Se compared to S, the examination of the theoretical spectra (Figure 4b) within the rich semi-extended family (see Figure 2) suggests another reason. Calculations confirm the robustness of the backbone bands within the family, but also provide evidence for a significant dependence of the 7^δ^ bond with the χ_3_ dihedral of the Sem-Met side-chain, which corresponds to different presentations of the heteroatom lone pair to the NH H-bond donor, the anti χ_3_ dihedral providing typically a 50 cm^−1^ increase in the IR red shift than its *g*+ counterpart (Figure 4b).

In conclusion, the significant change in the 7^δ^ H-bond strength when substituting Se to S should rather be ascribed to a change in side-chain conformation (from the 5_g+_-7^δ^-7 with S to the 5_a_-7^δ^-7 with Se), rather than to an intrinsic Se property. The lack of experimental data on the *same* forms for the S and Se compounds precludes experimental evidence for the relative H-bonding strengths for this backbone arrangement, but calculations confirm that, for the same conformation, the effect of substituting Se to S on the 7^δ^ H-bond strength remains modest, with a red shift increase remaining smaller than 15 cm^−1^ for *ag+a* side-chains (5a-7^δ^-7 conformations) and nearly inexistent for *ag+g+* side-chains.

### 5.3. General Considerations from NBO Analysis

**Figure 5:**
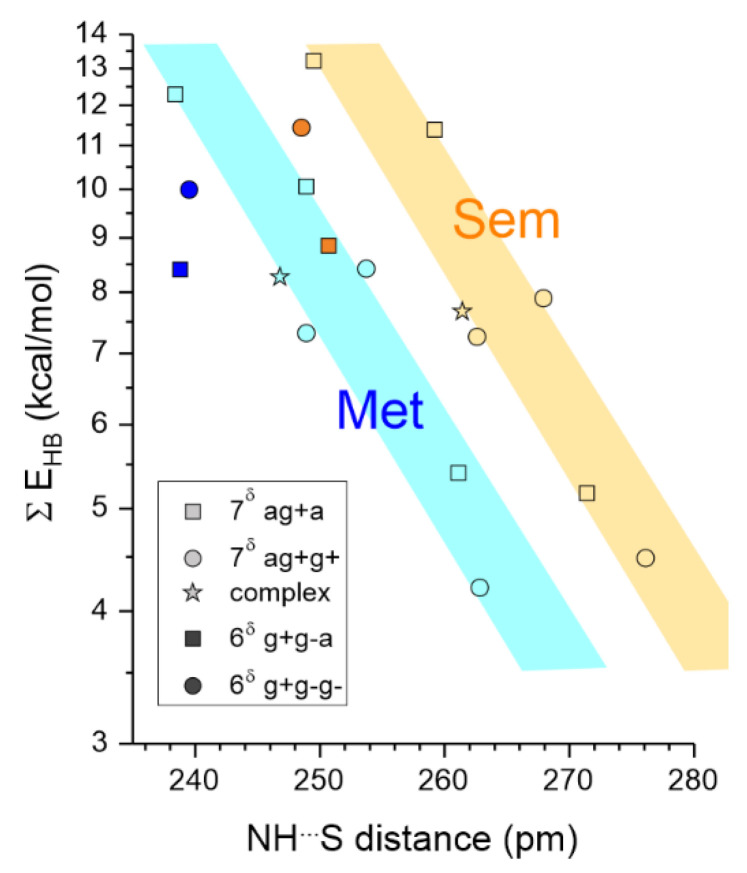
Semi-log plot of the stabilization energy ΣE_HB_ due to the NH⋯S H-bonds 5SEE (Tables S3 and S4) against the NH⋯S distance theoretically found for the 7^δ^ and 6^δ^ H-bonds (light and yellow symbols resp.) in the 5-7^δ^_g+/g*−*_-7_L_, 5-7^δ^/5_a_-π and 6^δ^-π_g+_-10 backbone conformations of the Ac-Sem-Met-Phe-NH_2_ dipeptides, with anti and gauche orientations of the Sem-Phe terminal dihedral χ_3_ (square and disk symbols resp.) together with the intermolecular MAA⋯S-SeMe_2_ complexes (stars) According to a previous NBO study of amide⋯amide interactions [29], in such plots the points aligned on the same trend illustrate comparable H-bond efficiency of the geometrical approach and enable to assess frustration or enhancement of the H-bond by its environment (see details in text).

The stabilization energy due to H-bonding was analyzed using the NBO tool for a selected series of conformations, capable of accounting for the observations, i.e., those mentioned in Figure 4 as well as the most stable extended form 5_a_-7^δ^/5_a_-π, together with, for reference, the Me-Se/S-Me⋯*trans*-methylacetamide intermolecular complex. Following an approach previously derived for amide–amide NH⋯OC interactions in peptides [29], which enables us to decouple the effect of the geometrical approach to that of the H-bond distance, the variation of the NBO stabilization (in a semi-log plot) with the NH⋯Se/S distance is drawn in Figure 5.

First of all, regarding the Se/S comparison, this plot demonstrates that for a given conformation, the S-compound exhibits a systematic shorter NH⋯Y distance of ~12 pm for the S compounds, readily assigned to the shorter van der Waals radius of S compared to Se [37]. In contrast, the stabilization energy between S and Se remains rather similar, with slight variations depending on the H-bond type. Se gives rise to a slightly stronger effect for the 7^δ^ and 6^δ^ bonds in *ag+a* and *g+g−g−* side-chains, respectively, in qualitative agreement with the spectral red shifts found theoretically between the Se and S compounds for the corresponding conformations. In the alternative side-chains, *ag+g+* and *g+g−a*, respectively, the difference is less obvious, again in agreement with the robustness of the 7^δ^ band in presence of the *ag+g+* side-chain (Figure 4b).

**Figure 6:**
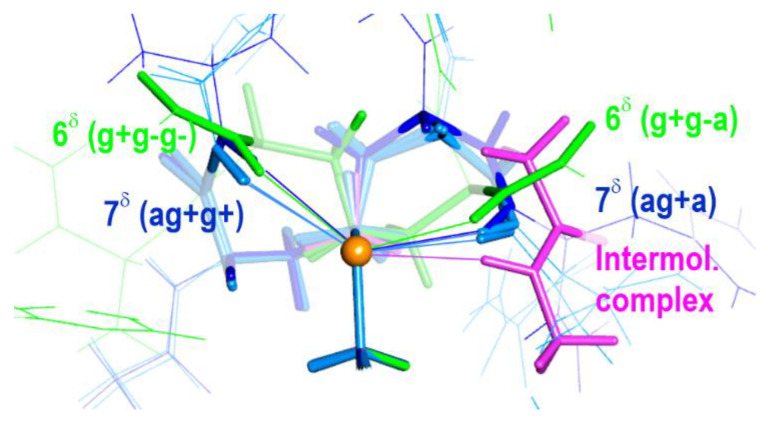
Comparison of the NH⋯Se approaches of the relevant conformations (5-7^δ^_g+/g—_7_L_ light blue, 5-7^δ^/5_a_-π dark blue and 6^δ^-π_g+_-10 green) of the Ac-Sem-Phe-NH_2_ compound, as found at the DFT-D level of theory. The terminal part of all the Sem side-chains was overlaid (Se atom in dark yellow), allowing to distinguish the effect of the χ_3_ dihedral orientation (side-chain dihedrals between parentheses). For the sake of clarity, front atoms were omitted and background atoms shaded. For reference, the structure of the MAA⋯S-SeMe_2_ intermolecular complex was also added. The same figure for the Ac-Met-Phe-NH_2_ compound, provided as Figure S1 in the Supplementary Material, illustrates the similarities between the two species.

Second, one notices that the 7^δ^ bonds of the Se/S compounds are fairly aligned on the same trend (colored strips in Figure 5), in a way similar to that reported in amide–amide NH⋯OC interactions within a set of peptide conformations, which share the same donor—acceptor geometrical approach. This suggests that the approaches provided by the 7^δ^ bonds (in blue in Figure 6) have a similar efficiency from the H-bonding point of view [29]. The difference in H-bond NH⋯Se-S distance between the *ag+g+* and *ag+a* side-chains in Sem or Met as well as between the semi-extended and extended forms should be thus ascribed to different covalent constraints in both the side-chain and the backbone, which, respectively, hamper or favor the formation of the H-bond. At this stage, comparison with the constraint-free MAA⋯S/SeMe_2_ intermolecular complexes is quite enlightening since the fact that the complex points in the Figure 5 plot (stars) lie close to the 7^δ^ lines demonstrates that the side-chain folding adopted for the formation of 7^δ^ bonds provides an H-bonding efficiency comparable to the best possible in the absence of constraints, i.e., that of the intermolecular complex (in magenta in Figure 6). This has clearly to be assigned to the increased flexibility of the Met-Sem side-chain provided by its two methylene groups, which enables the acceptor to find a favorable interaction geometry with the acceptor. Going further into the analysis of the 7^δ^ series, the positions of the points relative to that of the complex also provides insights into the role of covalent (intrinsic to the side-chains and the backbone) or non-covalent interactions (implicating both side-chain and backbone) in favoring or hampering the formation of a short H-bond for this approach. The long H- bond 7^δ^ distances (e.g., in extended conformations) correspond to frustrated structures with a good approach but whose covalent constraints hamper the bond shortening, in particular the presence of the 7_L_ fold on the C-terminus side of the molecule. Alternatively, short distances (e.g., in the 5_a_-7^δ^_g—_7L conformation) should be assigned to non-covalent interactions, between the side-chain terminal methyl group and the C-terminus carbonyl group, which tends to compress the NH⋯S distance and enhance the H-bond.

Third, the points representing the 6^δ^ bonds in Figure 5 fall below the 7^δ^ line, suggesting a less efficient geometrical approach for H-bonding (Figure 6 in green), possibly in link with the difference in the orientation of the peptide bond of the donor. However, the H-bond 6^δ^ distance shorter than in the intermolecular complexes again suggests that this H-bond benefits from constraints that tend to compress the H-bond within this approach, in particular through non-covalent interactions between the Sem-Met and Phe side-chains.

**Figure 7:**
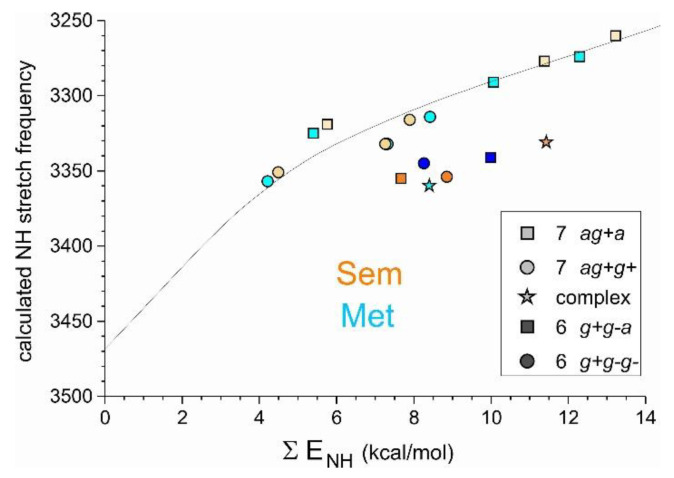
Calculated NH stretch frequency (scaled harmonic frequencies) vs. the total stabilization energy (noted ΣE_NH_), taking into account all the interactions that contribute to the electron delocalization towards the σ*_NH_ interaction, for the NH groups involved in an NH⋯Se-S interaction.

Besides these considerations, the NBO analysis also enables us to examine the correlation between the calculated NH stretch frequency and the total stabilization energy (noted ΣE_NH_), taking into account all the interactions that contribute to the electron delocalization towards the σ*_NH_ interaction (see Tables S3 and S4), namely beyond the NH⋯S H-bond, the accompanying C5 and π interactions in extended 5-7^δ^/5_a_-π and semi-extended 5-7^δ^/π-7_L_ conformations, respectively. The corresponding plot (Figure 7) demonstrates that in contrast to what was observed for the amide–amide NH⋯OC interactions, the dependence of the spectral shift with the delocalization-induced stabilization energy seems to depend significantly on the geometrical approach. A similar deviation is also found in the correlation between the NH stretch spectral shift and the population of the σ*_NH_ NBO (Figure S2).

Finally, the present data allow us to range the Sem-Met 7^δ^ and 6^δ^ H-bonds with respect to their 5^γ^ and 6^γ^ counterparts in the related seleno-cysteine and cysteine residues, which exhibit a shorter side-chain. Obviously, both NH stretch IR spectroscopy Cys [16,17,18] and NBO analysis Cys [17,18] provide evidence for weaker H-bonding in these covalently more constrained systems.

## 6. Conclusions

The present experimental and theoretical, conformation-resolved, comparative study of local side-chain–backbone NH⋯S/Se H-bonds of the Met-Sem residues illustrates the strong conformational similarities of the folding properties of these two residues. The conformational landscapes of the capped Met-Sem-Phe sequences studied are very similar: in both cases the same low lying conformations belonging to three different backbone families are present. The main differences stem from slightly different energetics among the families. Regarding the structures, the interaction distances with Se are larger, due to the larger van der Waals radius. However, both the wavefunction NBO analysis and the observation of NH stretch frequencies suggest that the interaction strength remains similar, with a slight difference in favor of one or the other, depending on the conformation considered.

Depending on the local side-chain–backbone H-bond considered (intra-residue 6^δ^ or inter-residue 7^δ^), the side-chain folding differ (with χ_1_,χ_2_ dihedrals of *g*+, *g*− and *a*, *g*+ types, respectively). For both foldings, however, the terminal -Me-Se/S-Me group can adopt a χ_3_ dihedral of *g* or of *a* type. The present NBO analysis shows that the latter orientation strongly affects the H-bond strength, essentially because it controls the NH⋯S/Se distance. As a matter of fact, 7^δ^ bonds with an *a* terminal side-chain orientation exhibit the stronger and the shorter H-bonds. The trend is reversed for the 6^δ^ bonds. Regarding the diversity of strengths, the 7^δ^ bonds are found to be strongly dependent upon the environment, the bonds being enhanced or frustrated depending on the conformation. A unique 6^δ^ bond was found, but this may be due to their appearance in a restricted number of conformations (β-turns). The spectral red shift they generate is significantly lower than that of the 7^δ^ bonds, which is interpreted in terms of a different and less effective approach of the H-bond acceptor S/Se to the backbone NH of the same residue, presumably because of the lesser flexibility of the side-chain. In the same line, for a similar total NBO stabilization energy, 6^δ^ bonds exhibit a lower red shift than their 7^δ^ counterparts (but comparable to the intermolecular MAA⋯S/SeMe_2_ complex), suggesting that the correlation between NBO stabilization energies and spectral shifts might not be as linear as previously reported [9] when the geometrical approaches are taken into account.

The present work also shows that the optical spectroscopic results, which at first glance suggest that Sem leads apparently to larger red shifts, must be confronted with an in-depth conformational analysis in order to reach valid comparisons: the apparent increase in red shift was just a consequence of a simple population transfer between S and Se, in which the side-chain terminal end is flipped from a *g*+ to an *a* orientation.

Finally, the optical spectroscopic results clearly show that experimental spectral NH stretch red shifts of up to 200 cm^−1^ can be reached for some conformations, illustrating the strength of the interaction, especially compared to amide–amide NH⋯OC bonds in natural peptides, whose largest red shift does not exceed 140 cm^−1^ [38]. In this regard, these present results support the conclusion of the recently published survey of NH⋯Se bonds in proteins, which emphasized the presence of strong NH⋯Se bonds in proteins [9] but suggests that they remain close in strength to the NH⋯S bonds.

## Data Availability

Details of quantum chemistry and assignment analyses are provided in the Supplementary Materials. Additional data that support the findings of this study, including structures, are available from the corresponding author upon reasonable request.

## Supplementary Materials

The following supporting information can be downloaded at: https://www.mdpi.com/article/10.3390/molecules27103163/s1, Table S1: Assignment table for the Ac-Sem-Phe-NH_2_ compound, Table S2: Assignment table for the Ac-Met-Phe-NH_2_ compound; Table S3: Relevant NBO data for Ac-Sem-Phe-NH_2_ and the intermolecular Se(Me)_2_⋯MMA; Table S4: Relevant NBO data for Ac-Met-Phe-NH_2_ and the intermolecular complex S(Me)_2_⋯MMA; Figure S1: Comparison of the geometrical approaches of Sem and Met in relevant conformations of Ac-Sem-Phe-NH_2_, Ac-Met-Phe-NH_2_ and of the intermolecular complexes S/Se(Me)_2_⋯MMA; Figure S2: NH stretch frequency vs. the total NBO stabilization energies ΣE_NH_.

## Sample Availability

The sample is commercial.
