# Supplementary material for: Selenium in Proteins: Conformational Changes Induced by Se Substitution on Methionine, as Studied in Isolated Model Peptides by Optical Spectroscopy and Quantum Chemistry
Source: Molecules. 2022 May 15;27(10):3163. doi: 10.3390/molecules27103163 (PMC9144663; doi:10.3390/molecules27103163)
Supplement: Supplementary file 1 [file molecules-27-03163-s001.zip › molecules-1716460-supplementary.pdf]

Selenium in proteins: Conformational changes induced by Se substitution on methionine, as studied in isolated model peptides by optical spectroscopy and quantum chemistry

Gildas Goldsztejn, Venkateswara Rao Mundlapati, Valérie Brenner, Eric Gloaguen, and Michel Mons*

Supplementary Materials

Table of contents

Table S1 : Assignment table for the Ac-Sem-Phe-NH_2 compound

Table S2: Assignment table for the Ac-Met-Phe-NH_2 compound

Table S3 : Relevant NBO data for Ac-Sem-Phe-NH_2 and the intermolecular complex $Se(Me)_2\cdots$MMA

Table S4 : Relevant NBO data for Ac-Met-Phe-NH_2 and the intermolecular complex $S(Me)_2\cdots$MMA

Figure S1 : Comparison of the geometrical approaches of Sem and Met in relevant conformations of Ac-Sem-Phe-NH_2, Ac-Met-Phe-NH_2 and of the intermolecular complexes S/$Se(Me)_2\cdots$MMA

Figure S2 : NH stretch frequency vs. population of the σ*NH NBO

Table S1 : Comparison of the experimental N-H stretch frequencies of the 4 conformers of **Ac-Sem-Phe-NH_2** with relevant theoretical data obtained for the lowest energy conformers of each backbone family (see Figure 2). Also given are, for each conformation: H-bond network and side chain orientations; relative enthalpies (ΔH, 0 K) and Gibbs free energies (ΔG, 300 K), both in kJ/mol. ; theoretical N-H stretch vibrational frequencies (cm^{-1}) and average and maximum deviations (<δ> and δ_{max} resp.) of the theoretical frequencies with respect to experimental frequencies of conformers A-D. These latter criteria can be used to guide the assignment, since typical discrepancy with experimental data is less than 20 cm^{-1} for free NH or NH···OC H-bonds. The best match conformations are indicated here in red in the eight last columns.

Experiment																
	Conformer			NH stretch frequencies (cm^{-1})												
	**A**			**3354**	**3381**	**3434**	**3520**									
	**D**			**3332**	**3389**	**3437**	**3519**									
	**B**			**3272**	**3381**	**3452**	**3520**									
	**C**			**3329**	**3422**	**3440**	**3539**									
Theory																
				NH stretch frequencies (cm^{-1})												
	ΔH (0 K) kJ/mol	ΔG (300 K) kJ/mol		NH_{Sem}	NH_2 symm.	NH_{Phe}	NH_2 antis.		**A**		**D**		**B**		**C**	
									<δ>	δmax	<δ>	δmax	<δ>	δmax	<δ>	δmax
**$6^{\delta}_{g-} - \pi_{g+} - 10$**	0.0	0.0		3331	3388	3448	3527		13	23	**5**	**11**	19	59	14	34
**$6^{\delta}_{a} - \pi_{g+} - 10$**	5.3	4.6		3354	3393	3447	3528		**8**	**13**	11	22	27	82	18	29
**$5_{g+} - 7^{\delta}_{g-} - 7_L$**	9.4	6.0		3332	3373	3453	3521		12	22	9	16	18	60	21	49
**$5_{g+} - 7^{\delta}_{g+} - 7_L$**	6.7	9.0		3316	3365	3460	3520		20	38	16	24	17	44	27	57
**$5_{g+} - 7^{\delta}_{a} - 7_L$**	13.3	9.6		3308	3399	3459	3522		23	46	15	24	16	36	20	23
**$5_{g+} - 7^{\delta}_{g-} - 7_D$**	5.8	8.0		3315	3360	3454	3524		21	39	17	29	17	43	26	62
**$5_a - 7^{\delta}_{g+} - 7L$**	8.5	9.0		3277	3378	3462	3528		29	77	25	55	**6**	**10**	32	52
**$5_a - 7^{\delta}_{g-} - 7L$**	11.4	10.0		3260	3374	3454	3516		31	94	27	72	**6**	**12**	39	69
**$5_a - 7^{\delta}_{a} - 7L$**	13.7	12.4		3249	3394	3458	3520		36	105	28	83	11	23	36	80
**$5_a - 7^{\delta}_{g+} - f$**	14.3	7.6		3274	3432	3447	3559		46	80	38	58	24	51	23	55
**$5_a – 7^{\delta}_{g-}\ 7_D$**	10.9	14.3		3267	3361	3451	3524		32	87	28	65	**7**	**20**	37	62
**$5_{g+} - 7^{\delta}/5_a – \pi$**	12.6	7.6		3351	3426	3444	3540		19	45	21	37	38	79	**8**	**22**
**$5_a - 7^{\delta}/5_a – \pi$**	16.9	11.5		3319	3425	3442	3538		26	44	18	36	30	47	**4**	**10**

Table S2 : Comparison of the experimental N-H stretch frequencies of the 4 conformers of **Ac-Met-Phe-NH_2** with relevant theoretical data obtained for the lowest energy conformers of each backbone family (see Figure 2). Also given are, for each conformation: H-bond network and side chain orientations; relative enthalpies (ΔH, 0 K) and Gibbs free energies (ΔG, 300 K), both in kJ/mol. ; theoretical N-H stretch vibrational frequencies (cm^{-1}) and average and maximum deviations (<δ> and δ_{max} resp.) of the theoretical frequencies with respect to experimental frequencies of conformers A-D. These latter criteria can be used to guide the assignment, since typical discrepancy with experimental data is less than 20 cm^{-1} for free NH or NH···OC H-bonds. The best match conformations are indicated here in red in the four last columns.

Experiment												
	Conformer			NH stretch frequencies (cm^{-1})								
	**A**			**3359**	**3385**	**3436**	**3518**					
	**B**			**3340**	**3359**	**3449**	**3518**					
Theory												
				NH stretch frequencies (cm^{-1})								
	ΔH (0 K) kJ/mol	ΔG (300 K) kJ/mol		NH_{Sem}	NH_2 symm.	NH_{Phe}	NH_2 antis.		**A**		**B**	
									**<δ>**	**δmax**	**<δ>**	**δmax**
**6^{δ}_{a} - π_{g+} - 10**	4.3	2.3		3360	3391	3445	3527		**6**	**9**	16	32
**6^{δ}_{g-} - π_{g+} - 10**	0.0	0.0		3341	3389	3444	3527		**10**	**18**	12	30
**5_{g+} - 7^{δ}_{g-} - 7_L**	10.0	6.0		3332	3375	3454	3522		15	27	**8**	**16**
**5_{g+} - 7^{δ}_{g+} - 7_L**	7.6	6.7		3314	3369	3459	3521		22	45	**12**	**26**
**5_{g+} - 7^{δ}_{a} - 7_L**	13.9	9.0		3321	3398	3457	3522		19	38	17	39
**5_{g+} - 7^{δ}_{g-} - 7_D**	6.6	7.6		3322	3361	3451	3525		21	37	**7**	**18**
**5_a - 7^{δ}_{g+} - 7L**	9.7	8.7		3291	3376	3460	3527		28	68	22	49
**5_a - 7^{δ}_{g-} - 7L**	12.1	9.8		3274	3379	3454	3517		28	85	23	66
**5_a - 7^{δ}_{a} - 7L**	13.2	10.1		3248	3397	3456	3522		37	111	35	92
**5_a - 7^{δ}_{g+} - f**	16.4	10.7		3330	3431	3435	3546		46	80	31	72
**5_a – 7^{δ}_{g-} 7_D**	11.7	6.4		3271	3361	3450	3524		33	88	19	69
**5_{g+} - 7δ/5_a – π**	11.7	6.4		3351	3426	3444	3540		19	45	27	66
**5_a - 7δ/5_a – π**	14.3	9.6		3319	3425	3442	3538		26	44	27	66

Table S3 : Detailed parameters of the NBO(i)→NBO(j) interactions at play in the 7^{δ} and 6^{δ} NH···Se H-bonds in several conformers of interest of the Ac-Sem-Phe-NH_2 capped dipeptide together with the NH···Se H-bond of the intermolecular *trans*-*N*-methylacetamide···$SeMe_2$ complex. Interaction energies E(2) are given in kcal/mol ; NBO occupancy is close to 2 for the donor NBOs considered. Depending on the conformation considered, several donor NBOs to the σ* NBO of the H-bonded NH have been found : namely the two Se lone pairs NBOs ($lp_{Se}(1)$ and $lp_{Se}(2)$), a π orbital of a C-C bond of the ring ($\pi_{CC\ Phe}$) as well as the two lone pairs NBOs ($lp_{OPhe}(1)$ and $lp_{OPhe}(2)$) of the neighbouring carbonyl O atom of the Phe residue in extended conformations

Se-compounds											
	Side chain	NH···Se distance (pm)	E(2) NBO stabilization energies (kcal/mol) for interaction from a donor NBO to the acceptor σ^*_{NH} NBO								
**Ac-Sem-Phe-NH_2**			donors								
conformation			$lp_{Se}(1)$	$lp_{Se}(2)$	$\pi_{CC\ Phe}$	$lp_{OPhe}(1)$	$lp_{OPhe}(2)$	ΣE_{HB}	ΣE_{NH}	σ^*_{NH} population (e)	Calc'd frequency
**5_a - 7^{δ}_{g+} - 7L**	ag+a	249.5	0.83	12.39				13.22	13.22	0.0684	**3274**
**5_a - 7^{δ}_{g-} - 7L**	ag+a	259.2	0.83	9.28	1.27			10.11	11.38	0.0631	**3291**
**5_{g+} - 7δ/5_a – π**	ag+a	271.4	0.26	4.72		0.19	0.59	4.98	5.17	0.0497	**3325**
**6^{δ}_a - π_{g+} - 10**	g+g-a	250.7	1.38	7.47				8.85	8.85	0.053	**3360**
**5_{g+} - 7^{δ}_{g-} - 7_L**	ag+g+	262.6	0.96	6.3				7.26	7.26	0.0558	**3332**
**5_{g+} - 7^{δ}_{g+} - 7_L**	ag+g+	267.9	1.02	5.5	1.37			6.52	7.89	0.0583	**3314**
**5_{g+} - 7δ/5_a – π**	ag+g+	276.1	0.56	2.66		0.29	0.98	3.22	4.49	0.0464	**3357**
**6^{δ}_{g-} - π_{g+} - 10**	g+g-g-	248.5	1.14	10.29				11.43	11.43	0.0567	**3341**
***t*-MMA···$SeMe_2$**											
**6^{δ}_a - π_{g+} - 10**		261.4	0.43	7.24				7.67	7.67	0.0515	**3355**

Table S4 : Detailed parameters of the NBO(i)→NBO(j) interactions at play in the 7^{δ} and 6^{δ} NH···Se H-bonds in several conformers of interest of the Ac-Met-Phe-NH_2 capped dipeptide together with the NH···Se H-bond of the intermolecular *trans-N*-methylacetamide···SMe_2 complex. Interaction energies E(2) are given in kcal/mol ; NBO occupancy is close to 2 for the donor NBOs considered. Depending on the conformation considered, several donor NBOs to the σ^* NBO of the H-bonded NH have been found : namely the two S lone pairs NBOs ($lp_S(1)$ and $lp_S(2)$), a π orbital of a C-C bond of the ring ($\pi_{CC\ Phe}$) as well as the two lone pairs NBOs ($lp_{OPhe}(1)$ and $lp_{OPhe}(2)$) of the neighbouring carbonyl O atom of the Phe residue in extended conformations

S-compounds											
	Side chain	NH···S distance (pm)	E(2) NBO stabilization energies (kcal/mol) for interaction from a donor NBO to the acceptor σ^*_{NH} NBO								
**Ac-Met-Phe-NH_2**			donors								
conformation			$lp_{Se}(1)$	$lp_{Se}(2)$	$\pi_{CC\ Phe}$	$lp_{OPhe}(1)$	$lp_{OPhe}(2)$	ΣE_{NH}	ΣE_{HB}	σ^*_{NH} population	Calc'd frequency
**5_a - 7^{δ}_{g+} - 7L**	ag+a	238.4	1.08	11.21				12.29	12.29	0.0684	**3274**
**5_a - 7^{δ}_{g-} - 7L**	ag+a	248.9	0.98	7.82	1.26			8.8	10.06	0.0631	**3291**
**5_{g+} - $7\delta/5_a - \pi$**	ag+a	261.1	0.36	4.07		0.23	0.74	4.43	5.4	0.0497	**3325**
**6^{δ}_a - π_{g+} - 10**	g+g-a	238.8	1.98	6.42				8.4	8.4	0.053	**3360**
**5_{g+} - 7^{δ}_{g-} - 7_L**	ag+g+	248.9	1.46	5.86				7.32	7.32	0.0558	**3332**
**5_{g+} - 7^{δ}_{g+} - 7_L**	ag+g+	253.7	1.53	5.4	1.49			6.93	8.42	0.0583	**3314**
**5_{g+} - $7\delta/5_a - \pi$**	ag+g+	262.8	0.78	2.44		0.22	0.77	3.22	4.21	0.0464	**3357**
**6^{δ}_{g-} - π_{g+} - 10**	g+g-g-	239.5	1.31	8.68				9.99	9.99	0.0567	**3341**
***t*-MMA···SMe_2**											
**6^{δ}_a - π_{g+} - 10**		246.8	0.71	7.55				8.26	8.26	0.0519	**3345**

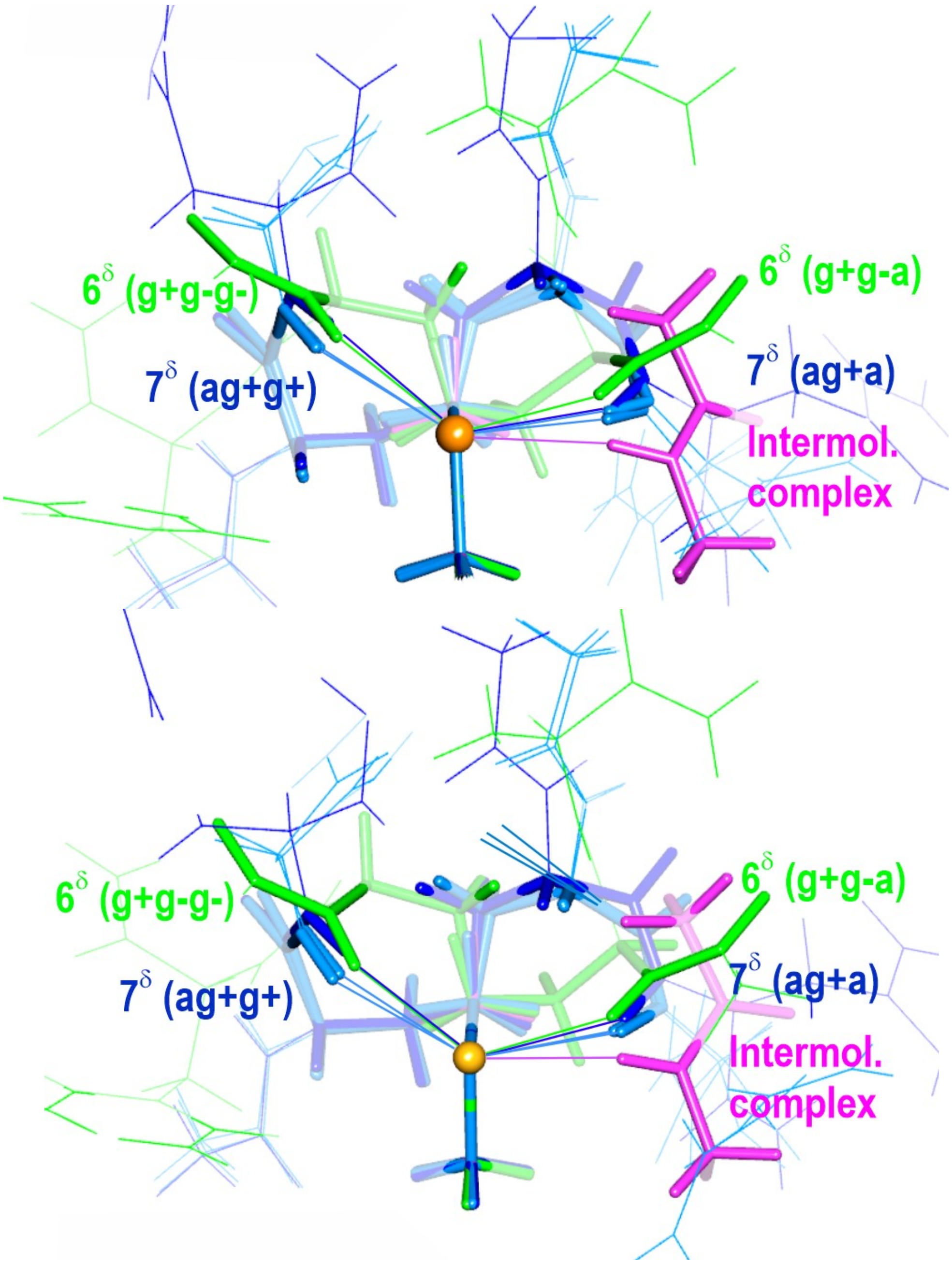

Ac-Sem-Phe-NH₂

Ac-Met-Phe-NH₂

Figure S1 : Comparison of the NH···S/Se approaches of the relevant conformations (5-$7^{\delta}_{g+/g-}$-7L light blue, 5-7^{δ}/5_a-π dark blue and 6^{δ}-π_{g+}-10 green) of the Ac-Sem-Phe-NH_2 (*top panel*) and Ac-Met-Phe-NH_2 (*bottom panel*) compounds, as found at the DFT-D level of theory. The terminal part of all the Sem/Met side chains have been overlaid (Se/S atom in yellow), allowing to distinguish the effect of the χ_3 dihedral orientation (side chain dihedrals between parentheses). For the sake of clarity, front atoms have been omitted and background atoms shaded. For reference, the structure of the intermolecular complex has also been added.

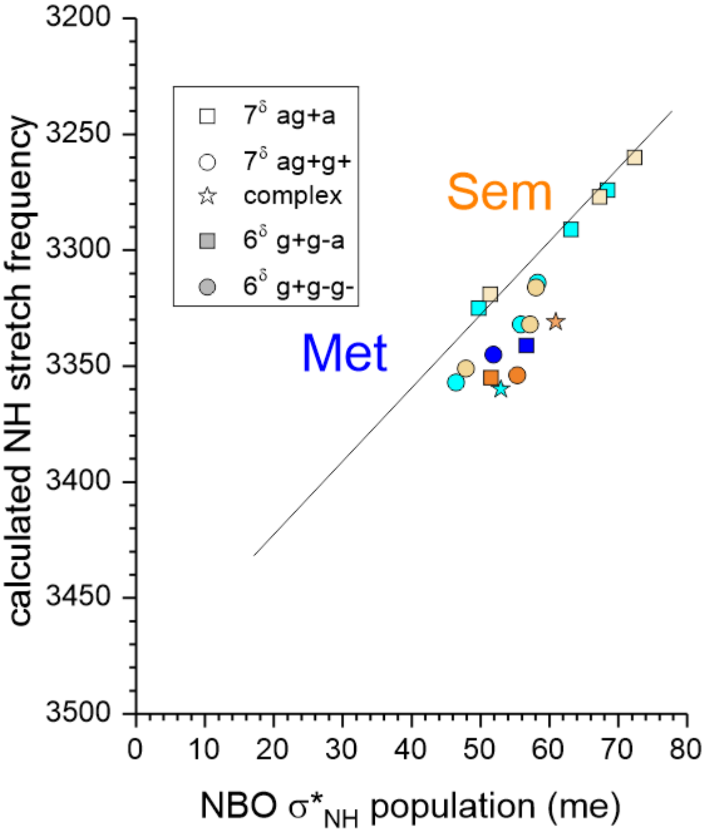

Figure S2 : Calculated NH stretch frequency (scaled harmonic frequencies) vs. the total stabilization energy (noted ΣE_{NH}), taking into account all the interactions that contribute to the electron delocalization towards the σ^*_{NH} interaction, for NH groups involved in a NH···S interaction, for all the conformations considered in the present analysis.
